# Polyunsaturated Fatty Acids of Marine Macroalgae: Potential for Nutritional and Pharmaceutical Applications 

**DOI:** 10.3390/md10091920

**Published:** 2012-08-24

**Authors:** Hugo Pereira, Luísa Barreira, Filipe Figueiredo, Luísa Custódio, Catarina Vizetto-Duarte, Cristina Polo, Eva Rešek, Aschwin Engelen, João Varela

**Affiliations:** Centre of Marine Sciences, University of Algarve, Faro 8005-139, Portugal; Email: hgpereira@ualg.pt (H.P.); lbarreir@ualg.pt (L.B.); fafigueiredo@ualg.pt (F.F.); lcustodio@ualg.pt (L.C.); cvduarte@ualg.pt (C.V.-D.); cris_polo10@hotmail.com (C.P.); eva.resek@gmail.com (E.R.); aengelen@ualg.pt (A.E.)

**Keywords:** marine macroalgae, nutrition, PUFA, EPA

## Abstract

As mammals are unable to synthesize essential polyunsaturated fatty acids (PUFA), these compounds need to be taken in through diet. Nowadays, obtaining essential PUFA in diet is becoming increasingly difficult; therefore this work investigated the suitability of using macroalgae as novel dietary sources of PUFA. Hence, 17 macroalgal species from three different phyla (Chlorophyta, Phaeophyta and Rhodophyta) were analyzed and their fatty acid methyl esters (FAME) profile was assessed. Each phylum presented a characteristic fatty acid signature as evidenced by clustering of PUFA profiles of algae belonging to the same phylum in a Principal Components Analysis. The major PUFA detected in all phyla were C_18_ and C_20_, namely linoleic, arachidonic and eicosapentaenoic acids. The obtained data showed that rhodophytes and phaeophytes have higher concentrations of PUFA, particularly from the *n*-3 series, thereby being a better source of these compounds. Moreover, rhodophytes and phaeophytes presented “healthier” ∑*n*-6/∑*n*-3 and PUFA/saturated fatty acid ratios than chlorophytes. *Ulva* was an exception within the Chlorophyta, as it presented high concentrations of *n*-3 PUFA, α-linolenic acid in particular. In conclusion, macroalgae can be considered as a potential source for large-scale production of essential PUFA with wide applications in the nutraceutical and pharmacological industries.

## Abbreviations

AA: arachidonic acid
ALA: α-linolenic acid
DHA: docosahexaenoic acid
DW: dry weight
EPA: eicosapentaenoic acid
FA: fatty acid
FAME: fatty acid methyl ester
GC-MS: gas chromatography-mass spectrometry
GLA: γ-linolenic acid
LA: linoleic acid
MUFA: monounsaturated fatty acid
PCA: principal component analysis
PUFA: polyunsaturated fatty acid
SFA: saturated fatty acid
VLCPUFA: very long chain polyunsaturated fatty acid

## 1. Introduction

Polyunsaturated fatty acids (PUFA) are of the utmost importance for human metabolism. They are the major components of cell membrane phospholipids [1], and may also be present in cellular storage oils [2]. In addition, PUFA are used in the biosynthesis of eicosanoids, hormone-like signaling molecules, which include thromboxanes, prostaglandins and leukotrienes [3]. Considering their fundamental role in metabolism, it comes as no surprise that beneficial properties have been attributed to PUFA, like antibacterial [4,5,6], anti-inflammatory [7,8], antioxidant [9], prevention of cardiac diseases [10], and inhibition of tumor progression [11,12]. Such properties are indicative of the potential of PUFA for nutraceutical and pharmaceutical purposes.

**Figure 1:**
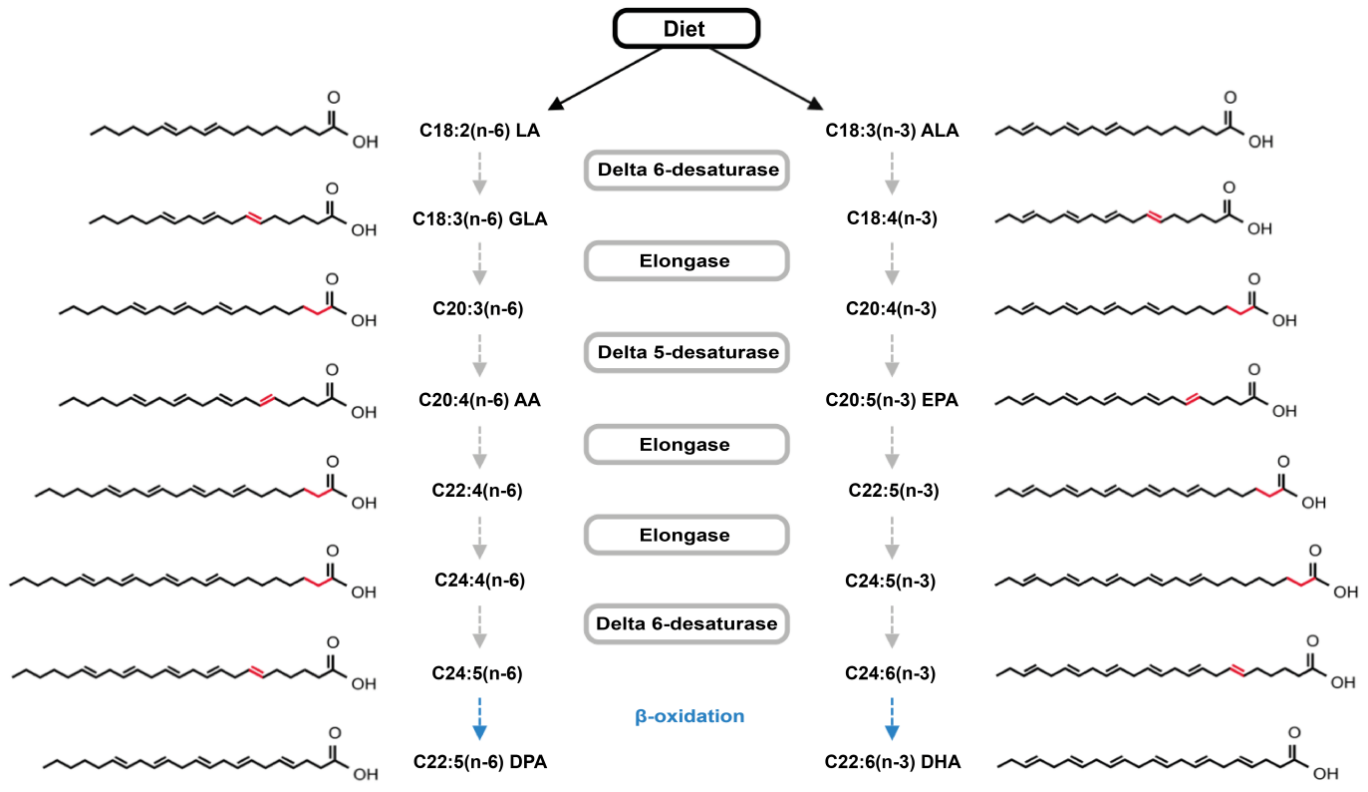
Schematic representation of the *n*-3 and *n*-6 fatty acid biosynthetic pathway with the enzymes responsible for each step of desaturation/elongation depicted in gray boxes. The partial β-oxidation that results in docosapentaenoic acid (DPA) or docosahexaenoic acid (DHA) formation is highlighted in blue. Differences occurring at each step are marked red in the chemical structure. Adapted from Marszalek and Lodish [19].

Almost all of the required long chain unsaturated fatty acids are synthesized by vertebrates through several elongation and desaturation steps [3,8] (Figure 1). The exceptions are α-linolenic acid (ALA) and linoleic acid (LA). These precursors for the biosynthesis of all other *n*-3 and *n*-6 PUFA cannot be synthesized by vertebrates and must, therefore, be present in diet, hence their classification as essential [13]. Humans can convert ALA to eicosapentaenoic acid (EPA) and docosahexaenoic acid (DHA); however, very long chain polyunsaturated fatty acids (VLCPUFA; >C_18_) are only synthesized to a limited extent: 8% and 21% for EPA and 4% and 9% for DHA in men and women, respectively [14,15,16,17]. Hence, in addition to the essential fatty acids, VLCPUFA must also be taken through dietary means or direct supplementation in order to meet with the European recommendations (European Food Safety Agency: EPA + DHA 250 mg/day) [18].

Synthesis of long chain *n*-3 and *n*-6 PUFA relies on the same enzymes and, generally, an increase in the amount of one of these essential fatty acids implies a decrease in the levels of the other, due to competition for the same metabolic enzymes [19]. This may cause an imbalance in the content of fatty acids (FA) and have a negative impact on human health. For example, a diet rich in *n*-6 PUFA may be linked to a prothrombotic and proaggregatory physiological state [20]. Consequently, the health promoting effects of these essential fatty acids are dependent on the maintenance of a proper balance between *n*-3 and *n*-6 PUFA [21]. 

The main known sources for ALA and LA are fish and vegetable oils. However, most westernized diets are unbalanced nowadays, due to the elevated consumption of meat- and vegetable oils-containing food products with high *n*-6 PUFA contents. Bearing in mind that fish is a declining resource [22] and that there is an increasing commercial interest in these long chain fatty acids [13], an alternative source of essential PUFA must be found. Despite their abundance, macroalgae are poorly exploited and, even though their total lipid content is usually low [23], they contain a high proportion of PUFA, combined with other interesting secondary metabolites (e.g., polysaccharides, vitamins, proteins). Together with the relative ease of cultivation and harvesting of macroalgae [24], this suggests that these marine photosynthetic organisms can be viable, sustainable sources of PUFA.

Hence, the objective of this work is to assess the potential of several Chlorophyta, Phaeophyta and Rhodophyta algae, found on the Algarve coast (Portugal), as a source of PUFA and/or specifically VLCPUFA. To the authors’ knowledge, the fatty acid composition of *Cladophora albida*, *Cladostephus spongiosus*, *Dictyota spiralis*, *Bornetia secundiflora* and *Asparagopsis armata* have yet to be published, thus providing an opportunity to widen the range of macroalgae strains with potential nutritional and/or pharmaceutical applications. Although the FA profiles of some of the target species in this work have already been characterized, intra-specific variability is common in macroalgae coming from different geographical locations, resulting in different FA profiles. This might be explained by exposure to diverse abiotic factors (e.g., temperature) that are known to influence the content of PUFA in algae [25,26].

## 2. Results and Discussion

### 2.1. FAME Concentration

**Figure 2:**
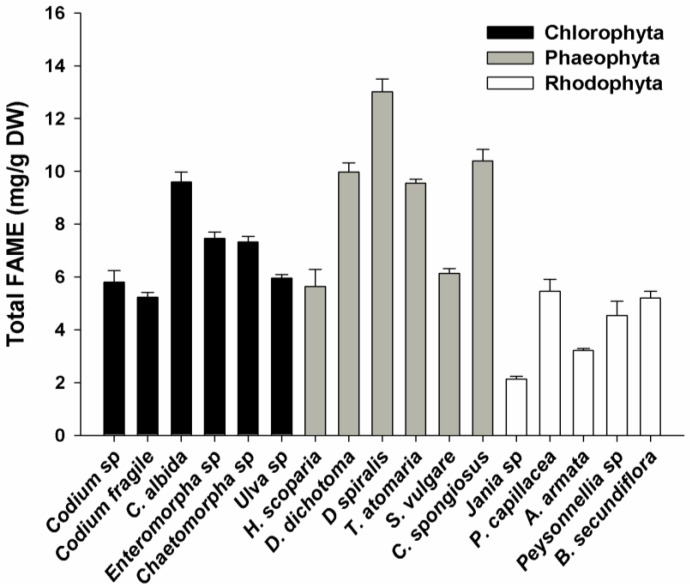
Total FAME concentration of macroalgae from three different phyla (Chlorophyta, Phaeophyta and Rhodophyta). Error bars represent the standard deviation from four replicates.

Total FAME concentration ranged from 2.1 in *Jania* sp. to 13.0 mg/g of dry weight (DW) in *Dictyota spiralis* (Figure 2). In the Chlorophyta algae, total FAME concentrations varied between 5.2 and 7.5 mg/g, except for *Cladophora albida* (9.5 mg/g). The Phaeophyta phylum presented significantly higher concentrations of total FAME than the other phyla (5.6–13.0 mg/g), namely in *Dictyota dichotoma*, *D. spiralis*, *Taonia atomaria* and *Cladostephus spongiosus* (*p* < 0.05). The lowest concentration of total FAME was recorded in the rhodophytes (*p* < 0.05), with all species presenting less than 5.5 mg/g. The relative concentration of lipids and corresponding FAME regarding each phylum is in accordance with previous reports [24].

### 2.2. FAME Profile

#### 2.2.1. Chlorophyta

The six species of the phylum Chlorophyta analyzed represent three different orders: Bryopsidales (*Codium* sp. and *C. fragile)*, Cladophorales (*Cladophora albida* and *Chaetomorpha* sp.), and Ulvales (*Enteromorpha* sp. and *Ulva* sp.). The most abundant FA in this phylum were palmitic (C16:0), myristic (C14:0), behenic (C22:0), palmitoleic (C16:1*n*-7), oleic (C18:1*n*-9c) and linoleic (C18:2*n*-6) acids (Table 1). These results are consistent with those found in the related literature for the same genera although for different species [27,28,29]. All species analyzed in this study presented considerably higher amounts of saturated fatty acids (SFA) than those reported previously. While in this study the SFA were more than 50% of the total detected FA, in the literature SFA relative amounts varied between 25% and 38% [27,28,29,30]. Conversely, the total concentration of PUFA of the Chlorophyta in this study ranged between 17% and 35%, significantly lower than those reported by other authors (37%–64%) [27,28,29,30]. Nevertheless, the C_18_ fatty acids were generally the dominant PUFA in all lipid profiles analyzed. Linoleic acid (LA; C18:2*n*-6) was the main PUFA of most chlorophytes. The only exception was *Ulva *sp., in which higher percentages of ALA (16%) were detected, in comparison to LA (5.7%). This ALA content is in accordance with earlier publications in which this FA was considered as characteristic of the Ulvales [24,26,27,28]. The lipid profiles of *Codium* sp. and *Chaetomorpha* sp. were the richest in terms of unsaturated fatty acids, while *Enteromorpha* sp*.* had the lowest PUFA content. *Codium* sp. was the only representative of this phylum in which γ-linolenic acid (GLA; C18:3*n*-6) was detected although it has been previously reported that species of the same genera contain this FA in minimal amounts (0.2%–2.3%) [27,28]. Both *Codium* species presented relatively high concentrations of *n*-3 hexadecatrienoic acid (C16:3*n*-3). Though this is a common FA within the *Codium* genus [28,29,31], the remaining analyzed species showed only trace amounts. EPA (20:5*n*-3) was detected in all chlorophytes at medium concentrations. In this phylum, EPA content ranged between 1% and 4% of the total fatty acid content and among the three analyzed phyla showed a trend similar to that of total PUFA, as also noticed by other authors [24]. DHA was only detected in *C. albida* (0.8%) and is fairly absent from this phylum, being reported in the literature at percentages lower than 1% [27,28,29].

**Table 1:** Fatty acid profile of the chlorophytes *Codium* sp, *C. fragile*, *Cladophora albida*, *Enteromorpha* sp., *Chaetomorpha* sp., and *Ulva* sp. Values are given as means of total FAME percentage ± standard deviation (*n* = 4). n.d., not detected.

Fatty acid (%)	*Codium *sp.	*Codium fragile*	*Cladophora albida*	*Enteromorpha *sp.	*Chaetomorpha sp.*	*Ulva *sp.
**C10:0**	0.42 ± 0.02	n.d.	n.d.	n.d.	n.d.	n.d.
**C12:0**	2.86 ± 0.15	1.53 ± 0.14	0.21 ± 0.01	0.39 ± 0.01	0.58 ± 0.02	n.d.
**C14:0**	4.42 ± 0.09	3.29 ± 0.18	12.48 ± 0.04	2.74 ± 0.04	21.74 ± 0.24	2.28 ± 0.03
**C15:0**	0.32 ± 0.01	n.d.	0.56 ± 0.02	0.90 ± 0.02	0.38 ± 0.01	0.49 ± 0.01
**C16:0**	32.75 ± 1.31	40.73 ± 0.83	33.04 ± 0.52	52.66 ± 0.80	33.24 ± 0.86	50.11 ± 0.34
**C17:0**	0.27 ± 0.01	n.d.	0.26 ± 0.01	0.28 ± 0.01	n.d.	n.d.
**C18:0**	1.34 ± 0.07	1.51 ± 0.06	1.28 ± 0.19	1.80 ± 0.01	1.03 ± 0.09	1.14 ± 0.02
**C20:0**	0.98 ± 0.10	1.01 ± 0.05	0.38 ± 0.01	2.08 ± 0.04	n.d.	n.d.
**C22:0**	6.28 ± 0.54	10.98 ± 1.06	0.75 ± 0.08	3.99 ± 0.10	1.00 ± 0.23	5.01 ± 0.78
**C24:0**	1.65 ± 0.15	3.32 ± 0.63	1.08. ± 0.01	n.d.	2.61 ± 0.17	n.d.
**∑ SFA**	**51.28 ± 1.44**	**62.37 ± 1.50**	**50.03 ± 0.56**	**64.85 ± 0.81**	**60.59 ± 0.94**	**59.04 ± 0.85**
**C16:1*n*-7**	3.34 ± 0.16	5.41 ± 0.17	13.90 ± 0.09	6.36 ± 0.07	2.83 ± 0.02	11.81 ± 0.14
**C18:1*n*-9c**	9.15 ± 0.04	1.49 ± 0.18	12.51 ± 0.02	9.08 ± 0.06	8.47 ± 0.03	5.51 ± 0.07
**C18:1*n*-9t**	0.89 ± 0.19	0.40 ± 0.94	0.79 ± 0.05	0.87 ± 0.10	0.30 ± 0.13	n.d.
**C20:1*n*-9**	0.21 ± 0.04	n.d.	0.18 ± 0.01	0.31 ± 0.01	0.24 ± 0.01	n.d.
**C22:1*n*-9**	n.d.	n.d.	0.35 ± 0.04	0.90 ± 0.02	n.d.	n.d.
**∑ MUFA**	**13.59 ±0.45**	**15.29 ± 0.97**	**27.73 ± 0.11**	**17.52 ± 0.14**	**11.84 ± 0.14**	**17.31 ± 0.15**
**C16:2*n*-6**	3.15 ± 0.10	1.40 ± 0.16	2.46 ± 0.01	0.83 ± 0.01	0.70 ± 0.02	n.d.
**C18:2*n*-6**	12.23 ± 0.48	9.21 ± 0.32	15.54 ± 0.22	10.04 ± 1.20	24.55 ± 0.32	5.65 ± 0.11
**C20:2*n*-6**	n.d.	n.d.	n.d.	n.d.	0.61 ± 0.01	n.d.
**C16:3*n*-3**	8.11 ± 0.39	5.92 ± 0.29	n.d.	0.50 ± 0.01	n.d.	n.d.
**C16:3*n*-6**	n.d.	n.d.	n.d.	n.d.	0.86 ± 0.01	n.d.
**C18:3*n*-3**	n.d.	n.d.	n.d.	n.d.	n.d.	16.51 ± 0.23
**C18:3*n*-6**	3.45 ± 0.16	n.d.	n.d.	n.d.	n.d.	n.d.
**C20:3*n*-6**	0.75 ± 0.03	0.91 ± 0.12	n.d.	n.d.	n.d.	n.d.
**C20:4*n*-6**	6.03 ±0.58	3.41 ± 0.20	1.37 ± 0.07	2.76 ± 0.09	n.d.	n.d.
**C20:5*n*-3**	1.40 ± 0.28	1.48 ± 0.17	2.02 ± 0.05	3.52 ± 0.06	0.85 ± 0.04	1.50 ± 0.04
**C22:6*n*-3**	n.d.	n.d.	0.86 ± 0.03	n.d.	n.d.	n.d.
**∑ PUFA**	**35.13 ± 0.91**	**22.34 ± 0.55**	**22.24 ± 0.24**	**17.64 ± 1.21**	**27.57 ± 0.33**	**23.65 ± 0.26**
**∑*n*-3**	**9.52 ± 0.48**	**7.40 ± 0.34**	**2.88 ± 0.05**	**4.02 ± 0.06**	**0.85 ± 0.04**	**18.00 ± 0.23**
**∑*n*-6**	**25.61 ± 0.78**	**14.93 ± 0.43**	**19.36 ± 0.23**	**13.62 ± 1.21**	**26.72 ± 0.32**	**5.65 ± 0.11**
**∑*n*-6/∑*n*-3**	**2.69**	**2.02**	**6.73**	**3.39**	**31.25**	**0.31**
**PUFA/SFA**	**0.68**	**0.36**	**0.44**	**0.27**	**0.46**	**0.40**

#### 2.2.2. Phaeophyta

The six Phaeophyta species analyzed belong to three different orders: Sphacelariales (*Halopteris scoparia* and *Cladostephus spongiosus*), Dictyotales (*Dictyota dichotoma*, *D. spiralis* and *Taonia atomaria*) and Fucales (*Sargassum vulgare*). Compared with the chlorophytes, the phaeophytes presented lower contents of SFA (*p* < 0.05), ranging from 30% to 45% of the total FAME detected (Table 2). This is consistent with other studies, which reported total SFA concentrations ranging from 20% to 44% for different phaeophytes [27,32]. Still, some exceptions could be found. For example, it has been reported a total SFA content of 66% and 53% for *Desmarestia viridis* and *Punctaria plantaginea*, respectively [27]. Similarly to other phaeophytes, myristic (C14:0) and palmitic (C16:0) fatty acids were the main SFA detected [27,29,30,32,33]. Total monounsaturated fatty acids (MUFA) concentrations (12%–30% of total FA) were significantly lower than those of SFA (*p* < 0.05) and the major MUFA detected were palmitoleic (C16:1*n*-7) and oleic (C18:1*n*-9c) acids. This is consistent with the profiles of the phaeophytes analyzed by other authors with the exception of *Homorsira banksii* in which the FA C17:1*n*-9 was a major MUFA in addition to the latter FA. This species was the only phaeophyte to present this FA both in the present study and in previously published literature [27,29,30,32,33]. The main PUFA detected in this phylum were C_18_ and C_20_ lipids, namely linoleic acid (C18:2*n*-6), arachidonic acid (AA; C20:4*n*-6) and EPA, which is in accordance with previous reports [27,32,33,34,35]. Still, in all studied species, the concentration of C_20_ PUFA was always generally higher than that of C_18_ PUFA, which is consistent with the typical profile of other phaeophytes [27,32,33]. The total PUFA concentration in these algae varied between 30% and 56% of the total FA, significantly higher than in green and red algae (*p* < 0.05). *H. scoparia*, *T. atomaria* and *C. spongiosus* were the species with the highest PUFA concentration (47%–57%). Accordingly, all phaeophytes displayed relatively high amounts of EPA (6%–14%), except for *D. spiralis* in which EPA was not detected. These relative EPA amounts are similar to those reported for other species of this phylum although higher values have been reported for several species, namely: *Scytosiphon lomentarius*, *Colpomenia sinuosa*, *Dictyosiphon foeniculaceus*, *Laminaria bongardiana*, *L. solidungula*, *Desmarestia muelleri*, *D. antartica* and *Myelophycus simplex* (19%–25%) [27,29,30,32,33]. Conversely, DHA was only detected in *H. scoparia*, *T. atomaria* and *S. vulgare* at low concentrations (0.8%–1.5% of total FA). In the literature, this FA is generally absent or exists in very small amounts in different phaeophytes [27,29,32].

**Table 2:** Fatty acid profile of the phaeophytes *Halopteris scoparia*, *Dictyota dichotoma*, *D. spiralis*, *Taonia atomaria*, *Sargassum vulgare*, and *Cladostephus spongiosus*. Values are given as means of total FAME percentage ± standard deviation (*n* = 4). n.d., not detected. n.a., not applicable.

Fatty acid (%)	*Halopteris scoparia*	*Dictyota dichotoma*	*Dictyota spiralis*	*Taonia atomaria*	*Sargassum vulgare*	*Cladostephus spongiosus*
**C10:0**	n.d.	n.d.	n.d.	n.d.	n.d.	n.d.
**C12:0**	n.d.	n.d.	n.d.	n.d.	n.d.	n.d.
**C14:0**	6.84 ± 0.20	15.42 ± 0.31	14.00 ± 0.13	7.07 ± 0.21	6.33 ± 0.02	7.40 ± 0.05
**C15:0**	0.43 ± 0.03	0.97 ± 0.06	0.73 ± 0.01	0.56 ± 0.01	0.62 ± 0.01	0.40 ± 0.01
**C16:0**	24.36 ± 0.45	24.75 ± 0.32	21.69 ± 0.22	25.41 ± 0.97	31.23 ± 0.24	21.33 ± 0.35
**C17:0**	0.37 ± 0.02	n.d.	0.23 ± 0.01	0.18 ± 0.01	0.22 ± 0.01	0.24 ± 0.01
**C18:0**	1.92 ± 0.10	2.85 ± 0.08	2.43 ± 0.04	1.04 ± 0.21	1.62 ± 0.11	1.15 ± 0.03
**C20:0**	0.98 ± 0.05	1.98 ± 0.12	1.12 ± 0.17	0.74 ± 0.09	0.94 ± 0.02	1.22 ± 0.04
**C22:0**	n.d.	n.d.	n.d.	0.48 ± 0.05	1.38 ± 0.11	n.d.
**C24:0**	n.d.	n.d.	n.d.	n.d.	n.d.	n.d.
**∑ SFA**	**34.89 ± 0.51**	**45.98 ± 0.47**	**40.20 ± 0.31**	**35.47 ± 1.02**	**42.34 ± 0.28**	**31.74 ± 0.35**
**C16:1*n*-7**	5.47 ± 0.09	15.49 ± 0.09	19.58 ± 0.12	8.09 ± 0.10	8.61 ± 0.11	5.72 ± 0.28
**C18:1*n*-9c**	5.57 ± 0.09	7.25 ± 0.06	7.57 ± 0.06	7.12 ± 0.21	6.08 ± 0.04	6.43 ± 0.18
**C18:1*n*-9t**	2.66 ± 0.25	1.24 ± 0.07	1.90 ± 0.04	0.97 ± 0.50	1.32 ± 0.02	n.d.
**C20:1*n*-9**	0.40 ± 0.02	0.31 ± 0.01	0.29 ± 0.03	0.30 ± 0.08	0.56 ± 0.01	n.d.
**C22:1*n*-9**	n.d.	n.d.	n.d.	0.85 ± 0.24	2.46 ± 0.06	n.d.
**∑ MUFA**	**14.09 ± 0.28**	**24.28 ± 0.13**	**29.34 ± 0.14**	**17.34 ± 0.61**	**19.03 ± 0.14**	**12.15 ± 0.33**
**C16:2*n*-6**	n.d.	0.44 ± 0.02	n.d.	n.d.	n.d.	n.d.
**C18:2*n*-6**	20.35 ± 0.14	5.55 ± 0.02	6.05 ± 0.10	10.08 ± 0.32	7.59 ± 0.02	23.14 ± 0.26
**C16:3*n*-3**	n.d.	n.d.	n.d.	n.d.	n.d.	n.d.
**C16:3*n*-6**	n.d.	n.d.	n.d.	n.d.	n.d.	n.d.
**C18:3*n*-3**	n.d.	n.d.	n.d.	n.d.	n.d.	n.d.
**C18:3*n*-6**	n.d.	2.63 ± 0.20	3.38 ± 0.05	1.71 ± 0.08	n.d.	3.10 ± 0.03
**C20:3*n*-6**	1.33 ± 0.09	2.60 ± 0.06	2.63 ± 0.14	2.14 ± 0.04	1.98 ± 0.29	1.62 ± 0.16
**C20:4*n*-6**	13.96 ± 0.36	11.46 ± 0.59	18.40 ± 0.21	18.64 ± 0.11	18.64 ± 0.04	16.43 ± 0.13
**C20:5*n*-3**	14.39 ± 0.25	6.57 ± 0.22	n.d.	13.55 ± 0.55	8.60 ± 0.12	11.46 ± 0.10
**C22:6*n*-3**	0.99 ± 0.86	n.d.	n.d.	0.84 ± 0.03	1.50 ± 0.07	n.d.
**∑ PUFA**	**51.01 ± 0.98**	**29.74 ± 0.67**	**30.46 ± 0.28**	**47.19 ± 0.65**	**38.63 ± 0.32**	**56.11 ± 0.35**
**∑*n*-3**	**15.37 ± 0.89**	**6.57 ± 0.22**	**n.d.**	**14.40 ± 0.55**	**10.10 ± 0.14**	**11.46 ± 0.10**
**∑*n*-6**	**35.64 ± 0.40**	**23.16 ± 0.63**	**30.46 ± 0.28**	**32.79 ± 0.35**	**28.52 ± 0.29**	**44.65 ± 0.33**
**∑*n*-6/∑*n*-3**	**2.32**	**3.52**	**n.a.**	**2.28**	**2.82**	**3.89**
**PUFA/SFA**	**1.46**	**0.65**	**0.76**	**1.33**	**0.91**	**1.77**

#### 2.2.3. Rhodophyta

The five representatives of the Rhodophyta phylum belong to five different orders, namely Corallinales (*Jania* sp.), Gelidiales (*Pterocladiella capillacea*), Bonnemaisoniales (*Asparagopsis armata*), Peyssonneliales (*Peyssonnelia* sp.) and Ceramiales (*Bornetia secundiflora*). Contrary to the other phyla, there was a greater variability in the lipid profile among the rhodophytes, as it can be seen by the total concentration of SFA that ranged between 39% (*Peyssonnelia* sp.) and 80% (*A. armata*). This variability was also reported by other studies in which SFA relative amounts ranged between 26% and 71% [27,32]. Similarly to what is described in the literature, the most abundant SFA in all strains studied were myristic and palmitic acids (Table 3) [27,29,30,32]. Total MUFA content of this phylum was lower than 10% of the total FA profile, except for *A. armata* and *B. secundiflora*, which presented slightly higher concentrations. Palmitoleic (C16:1*n*-7) and oleic (C18:1*n*-9c) acids were once more the main MUFA (Table 3). Other authors have described relatively higher amounts of MUFA in other rhodophytes although these were consistently the least representative of all FA [27,29,30,32]. Regarding PUFA, contrary to the other two phyla studied, linoleic acid (C18:2*n*-6) is not a major PUFA, reaching only 2% of the total FA. The most abundant PUFA in this phylum were AA (C20:4*n*-6) and EPA (20:5*n*-3), which are usually the most predominant FA in red algae [26,27,30,32,36]. However, as in SFA, there was a wide range in the total concentration of PUFA detected within species of this phylum: *A. armata* displayed only 5%, whereas in *Peyssonnelia* sp. PUFA content reached 52% of the total FA. Wide variability of PUFA content was also found by Graeve *et al*. [29], who reported a range in PUFA contents of 18%–63% in red algae from Arctic and Antarctic waters. Similarly, Li *et al.* [27] described PUFA contents ranging from 8% to 55% in rhodophytes from the Bohai Sea. Contrary to the other two phyla, in Rhodophyta, C_18_ are not the most representative PUFA. In fact, rhodophytes and phaeophytes exhibited considerably higher concentrations of C_20_ PUFA than chlorophytes [3,27,30,32,37]. In this study, except for *A. armata*, all strains exhibit considerably high amounts of EPA (15% and 27% of total FA). In the literature, Rhodophytes are commonly reported has good EPA producers, which suggests that red algae may be the best source of this nutritionally important fatty acid [27,29,30,32,38]. *Peyssonnelia* sp. was the only representative of this phylum in which DHA (C22:6*n*-3) was detected. In fact, this species presented significantly higher concentrations of this fatty acid than all algae studied (*p* < 0.05), reaching nearly 5% of the total FA (Table 3). DHA is often not found in red algae, or when present exists at low concentrations. Other authors have reported relative DHA amounts of 0.3% to 1.5% for several rhodophytes, which are significantly lower than those reported for *Peyssonnelia* in this study [29,38].

**Table 3:** Fatty acid profile of the rhodophytes *Jania* sp., *Pterocladiella capillacea*, *Asparagopsis armata*, *Peyssonnelia* sp., and *Bornetia secundiflora*. Values are given as means of total FAME percentage ± standard deviation (*n = *4). n.d., not detected.

Fatty acid (%)	*Jania *sp.	*Pterocladiella capillacea*	*Asparagopsis armata*	*Peyssonnelia *sp.	*Bornetia secundiflora*
**C10:0**	n.d.	n.d.	n.d.	n.d.	n.d.
**C12:0**	n.d.	n.d.	2.32 ± 0.09	n.d.	0.52 ± 0.01
**C14:0**	4.25 ± 0.08	9.68 ± 0.10	21.67 ± 0.11	5.50 ± 0.17	10.29 ± 0.01
**C15:0**	0.92 ± 0.01	0.41 ± 0.01	0.81 ± 0.02	0.60 ± 0.02	0.84 ± 0.17
**C16:0**	44.44 ± 0.29	47.94 ± 0.64	53.21 ± 0.52	29.50 ± 0.41	32.93 ± 0.75
**C17:0**	n.d.	0.39 ± 0.01	0.49 ± 0.02	0.61 ± 0.03	0.24 ± 0.01
**C18:0**	1.94 ± 0.06	2.21 ± 0.04	2.81 ± 0.16	2.94 ± 0.09	1.33 ± 0.22
**C20:0**	n.d.	n.d.	n.d.	n.d.	n.d.
**C22:0**	n.d.	n.d.	n.d.	n.d.	n.d.
**C24:0**	n.d.	n.d.	n.d.	n.d.	n.d.
**∑ SFA**	**51.56 ± 0.31**	**60.62 ± 0.65**	**81.31 ± 0.56**	**39.15 ± 0.46**	**46.14 ± 0.82**
**C16:1*n*-7**	2.38 ± 0.07	3.15 ± 0.09	4.87 ± 0.92	3.45 ± 0.07	12.75 ± 0.26
**C18:1*n*-9c**	2.54 ± 0.03	3.33 ± 0.01	2.78 ± 0.19	3.08 ± 0.02	2.13 ± 0.09
**C18:1*n*-9t**	2.01 ± 0.01	1.97 ± 0.02	6.34 ± 0.12	1.91 ± 0.07	3.78 ± 0.36
**C20:1*n*-9**	0.70 ± 0.01	n.d.	n.d.	0.42 ± 0.02	n.d.
**C22:1 *n*-9**	n.d.	n.d.	n.d.	n.d.	n.d.
**∑ MUFA**	**7.64 ± 0.08**	**8.45 ± 0.10**	**13.99 ± 0.94**	**8.87 ± 0.11**	**18.66 ± 0.46**
**C16:2*n*-6**	n.d.	n.d.	n.d.	n.d.	n.d.
**C18:2*n*-6**	2.37 ± 0.42	2.27 ± 0.05	n.d.	1.58 ± 0.08	1.64 ± 0.10
**C16:3*n*-3**	n.d.	n.d.	n.d.	n.d.	n.d.
**C16:3*n*-6**	n.d.	n.d.	n.d.	n.d.	n.d.
**C18:3*n*-3**	n.d.	0.93 ± 0.06	n.d.	n.d.	n.d.
**C18:3*n*-6**	n.d.	1.01 ± 0.04	n.d.	n.d.	2.53 ± 0.02
**C20:3*n*-6**	n.d.	1.14 ± 0.36	n.d.	n.d.	n.d.
**C20:4*n*-6**	12.99 ± 0.13	10.33 ± 0.09	1.79 ± 0.34	26.59 ± 0.31	3.78 ± 0.10
**C20:5*n*-3**	25.46 ± 0.53	15.26 ± 0.13	2.90 ± 0.15	18.52 ± 0.43	27.26 ± 0.64
**C22:6n-3**	n.d.	n.d.	n.d.	4.86 ± 0.18	n.d.
**∑ PUFA**	**40.81 ± 0.69**	**30.94 ± 0.40**	**4.70 ± 0.38**	**51.98 ± 0.53**	**35.20 ± 0.66**
**∑*n*-3**	**25.46 ± 0.53**	**16.19 ± 0.14**	**2.90 ± 0.15**	**23.38 ± 0.47**	**27.26 ± 0.64**
**∑*n*-6**	**15.35 ± 0.44 **	**14.74 ± 0.37**	**1.79 ± 0.34**	**28.60 ± 0.25**	**7.94 ± 0.14**
**∑*n*-6/∑*n*-3**	**0.60**	**0.91**	**0.62**	**1.92**	**0.29**
**PUFA/SFA**	**0.79**	**0.51**	**0.06**	**1.33**	**0.76**

#### 2.2.4. Multivariate Analysis

**Figure 3:**
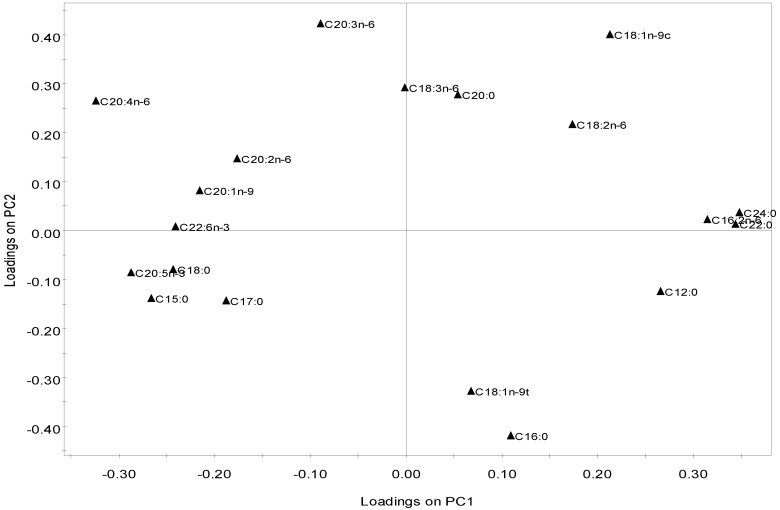
PCA plot of the macroalgae fatty acid composition profiles showing the loadings on PC1 and PC2, representing 26.0% and 20.5% of the total variance of the data, respectively.

**Figure 4:**
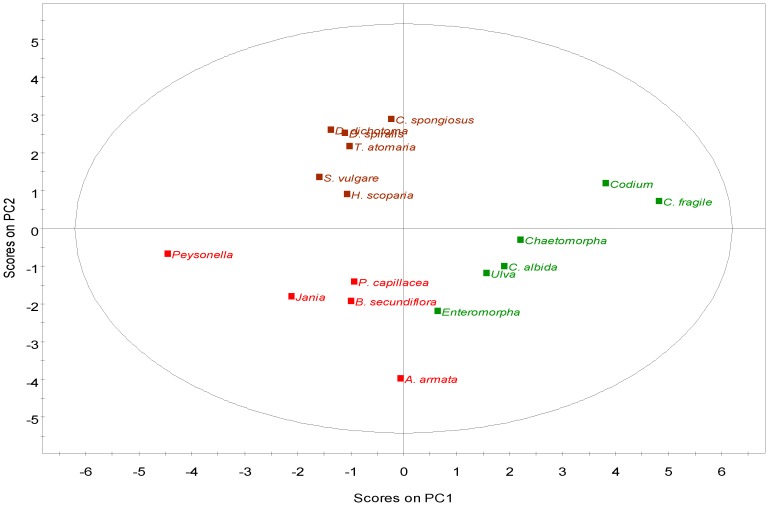
PCA of the fatty acid composition of macroalgae showing the data scores labeled by phylum. Green—Chlorophyta; Brown—Phaeophyta; Red—Rhodophyta.

Principal Components Analysis (PCA) was performed on the 21 detected fatty acids as a proportion of total fatty acid content, to assess the relationship between the 17 strains belonging to the three different phyla. The levels of capric (C10:0), myristic, hexadecatrienoic (*n*-3 and *n*-6) and ALA were removed from the multivariate analysis due to their low variation, therefore promoting a more reliable analysis. The first two components explain 46.6% of the total variation, 26% for PC1 and 20.5% for PC2 (both significant). In the loading scatter plot of the variables (FA; Figure 3), AA, EPA, DHA, pentadecylic (C15:0), behenic (C22:0), lignoceric (C24:0) and hexadecadienoic (C16:2*n*-6) acids were the most discriminant variables along PC1, while palmitic (C16:0), oleic (C18:1*n*-9c), elaidic (C18:1*n*-9t) and dihomo-γ-linolenic (C20:3*n*-6) acids were the main discriminant FA along PC2. In this plot, FA are distributed according to their saturation: most SFA are placed on the upper quadrants while most PUFA grouped in the lower left quadrant. In the scores plot, species clustered according to their phylum (Figure 4), suggesting that each phylum has a distinct FA profile and supporting earlier evidence that lipid composition may be a biochemical marker for each taxonomic group [24,36,39]. Besides its evident application in phylogenic studies, this feature may help to assign different algae to distinct diets. In this sense, the placement of the rhodophytes and phaeophytes in the left quadrants, and of all chlorophytes in the right quadrants, indicate that the first two phyla display a lipid profile clearly enriched in pentadecylic, stearic, EPA, DHA, AA and eicosenoic (C20:1) acids. Within the Chlorophyta phylum, *Codium* strains are located further to the right due to their increased content in *n*-3 hexadecatrienoic (C16:3*n*-3), behenic (C22:0) and lignoceric (C24:0) acids. The Rhodophyta and Phaeophyta species are separated only along PC2, with the relative proportions of AA and dihomo-g-linolenic acid as the main discriminant factors (Figure 4). 

### 2.3. Nutritional and Pharmaceutical Applications

PUFA are vital components in human nutrition and are known to have several beneficial effects for human health. A diet intake of PUFA, including both *n*-3 and *n*-6 fatty acids, is known to modulate inflammatory processes among other cell functions. Although many of the species analyzed in this work displayed high amounts of SFA, some Phaeophyta and Rhodophyta species exhibited higher concentrations of PUFA, and PUFA/SFA ratios higher than 1 (*H. scoparia*, 1.46; *T. atomaria*, 1.33; *C. spongiosus*, 1.77; *Peyssonnelia *sp., 1.33). The lowest PUFA/SFA ratios were observed in algae from the phylum Chlorophyta (0.27–0.68). It appears that this phylum has a lower potential, comparing to the other two phyla studied, as a nutritional source of PUFA for human consumption. The results presented herein are in agreement with previous studies in which rhodophytes and phaeophytes displayed higher concentrations of unsaturated fatty acids as compared with chlorophytes [24]. However, not all PUFA are associated with the promotion of health benefits. For example, in the inflammation process, eicosanoids derived from *n*-6 PUFA are generally considered as pro-inflammatory or as promoters of other cell harmful effects, whereas *n*-3 PUFA derivatives are considered less inflammatory or even anti-inflammatory [8,40,41]. Since the biosynthetic pathway of these fatty acids relies on the same enzymes for *n*-3 and *n*-6 PUFA, the health promoting effects are dependent on the *n*-6/*n*-3 ratio of PUFA obtained through diet. The World Health Organization (WHO) recommends a ∑*n*-6/∑*n*-3 ratio lower than 10 [21,23]. In this study, almost all algae can be considered as a good source of dietary PUFA, since they showed ratios ranging between 0.29 and 6.73. The exception was *Chaetomorpha* sp., in which the ∑*n*-6/∑*n*-3 ratio was the highest from all the studied species (31.25) and in *D. spiralis* in which no *n*-3 fatty acids were detected. 

Besides an appropriate nutritional profile, these macroalgae can also be exploited for pharmaceutical purposes. Many of the PUFA detected throughout this work are considered powerful molecules against several diseases and are already used in different biomedical applications. For example, several reports suggest that *n*-3 fatty acids, mainly EPA and DHA, may have a significant potential in the treatment of autoimmune and inflammatory diseases [42]. In this study, Rhodophyta was the phylum with the highest percentage of *n*-3 fatty acids (16%**–**27% of total FAME), followed by Phaeophyta (0%**–**15%), in which significant amounts of *n*-3 were also present. Aside from *Ulva* sp. that had 18% of *n*-3 FAME, Chlorophyta macroalgae presented the lowest values of *n*-3 fatty acids (1%**–**9%). Conversely, the detected *n*-6 fatty acids were lower in rhodophytes (8%**–**15%), due to the low concentration of linoleic acid, except for *Peyssonnelia* sp., where *n*-6 concentration was approximately 28% of total FAME. Phaeophytes showed the highest contents of *n*-6 fatty acids (23%**–**44%), whereas chlorophytes presented mid-range values (6%**–**27%). Considering the absolute concentrations of PUFA in the various species tested, *Ulva* sp., *T. atomaria*, *C. spongiosus*, *Peyssonnelia* sp. and *B. secundiflora* possess the highest contents of *n*-3 PUFA, 1.07, 1.38, 1.19, 1.06 and 1.42 mg/g, respectively. Apart from *Ulva* sp., in which ALA dominated, the *n*-3 profile of the remaining strains was essentially composed of EPA. DHA was not a major PUFA in any of the algae studied in this work. Nevertheless, *Peyssonnelia* sp. exhibited a relatively high content of DHA, 0.22 mg/g of dry biomass, coupled with an EPA concentration of 0.84 mg/g. A variety of potential applications are described for EPA and DHA, which hold significant potential for pharmaceutical purposes, namely cancer treatment, asthma, psoriasis, rheumatoid arthritis, antibiotic, inflammatory bowel disease, depression, allergies, cardiovascular diseases, among others [1,20]. More recently PUFA proved to have a strong potential in drug delivery; in addition to the described cytotoxicity of a few PUFA, PUFA enable a more efficient penetration of specific molecules through the cell membranes of tumor cells, due to their unique lipophilic characteristics [43]. In fact, several studies show that tumor cells display faster PUFA intake than normal cells, as demonstrated for the conjugated taxoid DHA-paclitaxel [12]. 

The nutritional and pharmaceutical benefits of PUFA, however, contrast with the increasing difficulty in finding sustainable sources of *n*-3 VLCPUFA, which traditionally were obtained from fish and fish oil. Declining fish stocks caused by decades of overfishing [44] makes ever more urgent to find non-traditional alternatives for the western world. As VLCPUFA are usually absent from terrestrial higher plants [45], traditional crops can also be excluded as viable sources of these FA. Though this deficiency can be overcome by applying genetic engineering, transgenic foods are not always well accepted by the general public. Therefore, *n*-3 VLCPUFA are typically associated with marine organisms, and algae, as the basis of the marine trophic chain, come out as a very promising source of VLCPUFA. In fact, large scale farming of marine algae has been accomplished successfully for hundreds of years [46]. Approximately 220 algal species are currently cultivated and harvested all over the world for different purposes [47]. Though mostly used as food for human consumption, particularly in Asia, macroalgae are also the primary source of hydrocolloids such as agar, carrageenan and alginate, which have numerous industrial applications, such as gelling, stabilizing or binding agents [47,48,49]. The next step could well be the sustainable exploitation of marine macroalgae as alternative sources of VLCPUFA, not only in Asia, but also in the western world.

## 3. Experimental Section

### 3.1. Sampling and Processing of Macroalgae

Macroalgae biomass was collected in May 2010 at beaches throughout the Algarve coast, namely: Vila Real de Santo António, Faro, Albufeira and Odeceixe. Overall, seventeen species belonging to the phylum Chlorophyta (*Codium* sp., *C. fragile*, *Cladophora albida*, *Enteromorpha* sp., *Chaetomorpha* sp. and *Ulva* sp.), Phaeophyta (*Halopteris scoparia*, *Dictyota dichotoma*, *D. spiralis*, *Taonia atomaria*, *Sargassum vulgare* and *Cladostephus spongiosus*) and Rhodophyta (*Jania* sp., *Pterocladiella capillacea*, *Asparagopsis armata*, *Peyssonnelia* sp. and *Bornetia secundiflora*) were collected. Tissues were selected and separated to avoid cross-contamination, rinsed with freshwater, freeze dried, homogenized to powder and stored at −20 °C until further analysis.

### 3.2. FAME Preparation

Lipids and free fatty acids were converted to the corresponding FAME, according to a modified protocol of Lepage and Roy [50]. This method is based on the direct transesterification with acetyl chloride/methanol, followed by direct extraction of the lipidic phase into hexane. Briefly, 0.1 g of algal biomass was weighed and treated with 1.5 mL of derivatization solution (methanol/acetyl chloride, 20:1, v/v), in reaction vessels. The biomass was disrupted with an IKA Ultra-Turrax disperser and afterwards 1 mL of hexane was added and the mixture heated for 1 hour at 100 °C. After cooling in an ice bath, 1 mL of distilled water was added and the organic phase was removed and dried with anhydrous sodium sulfate. The extracts were then filtered and stored at −20 °C until further analysis.

### 3.3. Determination of FAME Profile by GC-MS

FAME were analyzed on an Agilent GC-MS (Agilent Technologies 6890 Network GC System, 5973 Inert Mass Selective Detector) equipped with a DB5-MS capillary column (25 m × 0.25 mm internal diameter, 0.25 µm film thickness, Agilent Tech) using helium as carrier gas. Samples were injected at 300 °C and the temperature profile of the GC oven was 60 °C (1 min), 30 °C min^−1^ to 120 °C, 5 °C min^−1^ to 250 °C, and 20 °C min^−1^ to 300 °C (2 min). For the identification and quantification of FAME, the total ion mode was used. A “Supelco^®^ 37 Component FAME Mix” (Sigma-Aldrich, Sintra, Portugal) was used as a standard and separate calibration curves were generated for each of the FAME in this standard. When there was no standard available, the calibration curve of the most similar FAME in terms of structure was used. Values were expressed as mg/g of dry weight.

### 3.4. Statistical Analysis

Obtained results are presented as mean ± standard deviation; all analyses were performed in quadruplicate. Differences between species or phyla were assessed using analysis of variance (one-way ANOVA) while differences between FAME groups were determined using the *t*-test for independent samples. Significant differences were considered when *p* < 0.05 by means of the statistical program StatSoft STATISTICA (release 7.0). PCA was used to compare the FAME profiles of different macroalgae, as previously published by other authors [24,36,39]. PCA is used to transform an original set of potentially correlated variables into a reduced set of uncorrelated variables that are called principal components. These components are obtained in order of decreasing importance. The first principal component explains the most variance; the second principal component explains the next degree of variance. For each sample, PCA calculates a “score” and two-dimensional plots of the scores for the first two principal components, which may reveal clusters and trends in data. Principal component analysis was performed in Umetrics SIMCA-P software (release 12.0.1).

## 4. Conclusions

VLCPUFA are well known bioactive molecules with important nutritional and pharmaceutical applications. The FA content of the macroalgae studied displayed similar signatures within species of the same phylum. However, when chlorophytes, phaeophytes and rhodophytes were compared among each other, distinct FA profile clusters per phylum were observed. Representatives of the Rhodophyta and Phaeophyta had significantly higher concentrations of VLCPUFA, namely the C_20_, AA and EPA. In particular, *Ulva* sp. had a lipid profile particularly enriched in the *n*-3 fatty acid ALA (16%), whereas *T. atomaria*, *C. spongiosus* and *B. secundiflora* presented the highest contents of EPA (>1 mg/g). DHA was not a dominant VLCPUFA in the studied species, although *Peyssonnelia* sp had 0.22 mg/g of this FA, nearly 5% of the total FAME. In combination with 0.84 mg/g of EPA the latter macroalga makes an excellent and balanced source of *n*-3 VLCPUFA. Taken together, these results suggest that most macroalgae may provide human beings with beneficial FA in their diets if used as nutrient sources in food products commonly consumed in the western world.
